# The predictors of short and long term urinary continence recovery after laparoscopic radical prostatectomy: a single cancer center report in China

**DOI:** 10.1186/s12957-024-03425-2

**Published:** 2024-06-06

**Authors:** Lei Liu, Shukui Zhou, Dandan Song, Zeng Li, Shengke Yang, Yi Wu, Guiying Zhang, Duocai Tang, Junfeng Liu, Hong Liao, Chuan Zhang

**Affiliations:** 1 Department of Urology, People’s Hospital of Dayi County, Chengdu, 611300 China; 2 Department of Urology, Sichuan Clinical Reasearch Center for Cancer, Sichuan Cancer Hospital & Institute, Sichuan Cancer Center, Affiliated Cancer Hospital of University of Electronic Science and Technology of China, Chengdu, 610041 China

**Keywords:** Laparoscopic radical prostatectomy, Urinary continence, Predictors, Magnetic resonance imaging, Membranous urethral length

## Abstract

**Purpose:**

To evaluate the predictors for short and long term urinary continence (UC) recovery after laparoscopic radical prostatectomy (LRP) from clinical and oncological variables.

**Methods:**

We retrospectively collected data from 142 prostate cancer patients who underwent LRP between September 2014 and June 2021 at a tumor specialist diagnosis and treatment center in China. The rate of post-prostatectomy incontinence (PPI) was evaluated from immediate and at 3, 6 and 12 mo after LRP, and UC was defined as the use of no or one safety pad. Sixteen clinical and oncological variables were analyzed by univariate and multivariate regression analysis to determine whether they were associated with short (3 mo) or long term (12 mo) UC recovery after LRP.

**Results:**

After eliminating patients who were lost to follow-up, 129 patients were eventually included. The mean ± SD age was 68 ± 6.3 years. The UC rates of immediate, 3, 6 and 12 mo after the operation were 27.9%, 54.3%, 75.2% and 88.4%, respectively. Multivariate analyses revealed that membranous urethral length (MUL) was a protective predictor of UC after catheter extraction(*P* < 0.001), and at 3 mo (*P* < 0.001), 6 mo (*P* < 0.001) and 12 mo (*P* = 0.009) after surgery.

**Conclusion:**

MUL is a significant independent factor that can contribute to short and long term UC recovery post-LRP, which may assist clinicians and their patients in counseling of treatment.

## Introduction

Prostate cancer (PCa) is the most common malignancy of the urogenital system in American men [1]. PCa is one of the fastest growing malignancies in China, and by 2022, its incidence in China is expected to be 0.58 times than that of the USA, but the death rate is 1.62 times higher [2, 3]. Laparoscopic radical prostatectomy (LRP) and robot-assisted radical prostatectomy (RARP) are the standard surgical treatments for clinical localized PCa. For RP, there are three main goals known as the trifecta: cancer control, urinary function and sexual function [4]. Urinary incontinence (UI) is closely associated quality of life after LRP. A meta-analysis suggested that UI at 3 and 12 mo after RARP were 14–35% and 4–31%, respectively, which defined continence as wearing no or one safety pad [5]. Although the physiology and mechanisms of post-prostatectomy incontinence (PPI) are complex, several studies have reported that PPI may be related to age, body mass index (BMI), Charlson Comorbidity Index (CCI), D’Amico risk group, pelvic lymph node dissection (PLND) intravesical prostatic protrusion (IPP) or membranous urethral length (MUL) [6, 7]. However, the literature on the predictive value of different risk factors for PPI often shows conflicting results. In this study, we incorporated patient general conditions, tumor and multiparametric magnetic resonance imaging (mpMRI) features, aimed to investigate the predictive factors of UC recovery after LRP at a single cancer center of China.

## Materials and methods

### Patients

We obtained data from 142 PCa patients who were performed LRP by the same surgeon between September 2014 and June 2021 at a tumor specialist diagnosis and treatment center in China. Pelvic lymph node dissection is routinely performed in PCa patients with a > 5% risk of pelvic lymph node metastasis according to the Briganti nomogram [8]. Patient data were collected retrospectively; 13 patients were excluded because of loss to follow-up, leaving 129 eligible patients for further analysis. UC was defined as the use of no or one safety pad per day. We defined the time of Foley catheter removal as immediate UC recovery (usually 2–3 wk after surgery) [9], 3 mo UC was defined as short term, and 12 mo UC as long term [10].

### Clinical and pathological parameters

Patient-related parameters were age, BMI, smoking history, complication with chronic diseases, and transurethral resection of prostate (TURP) history or neoadjuvant therapy. Tumor-related parameters were prostate-specific antigen (PSA), clinical T stage, D’Amico risk group, and postoperative International Society of Urological Pathology (ISUP) grade. PSA was divided into three groups: < 10 ng/ml, 10–20 ng/ml and > 20ng/ml. Clinical T stage was divided into three groups: ≤ T2a, T2b, ≥ T2c. Operation-related parameters included time of operation, pelvic lymph node dissection, and neurovascular bundle preservation. MRI-based anatomical parameters were IPP, prostate volume, MUL and bladder neck diameter.

### MRI

**Fig. 1:**
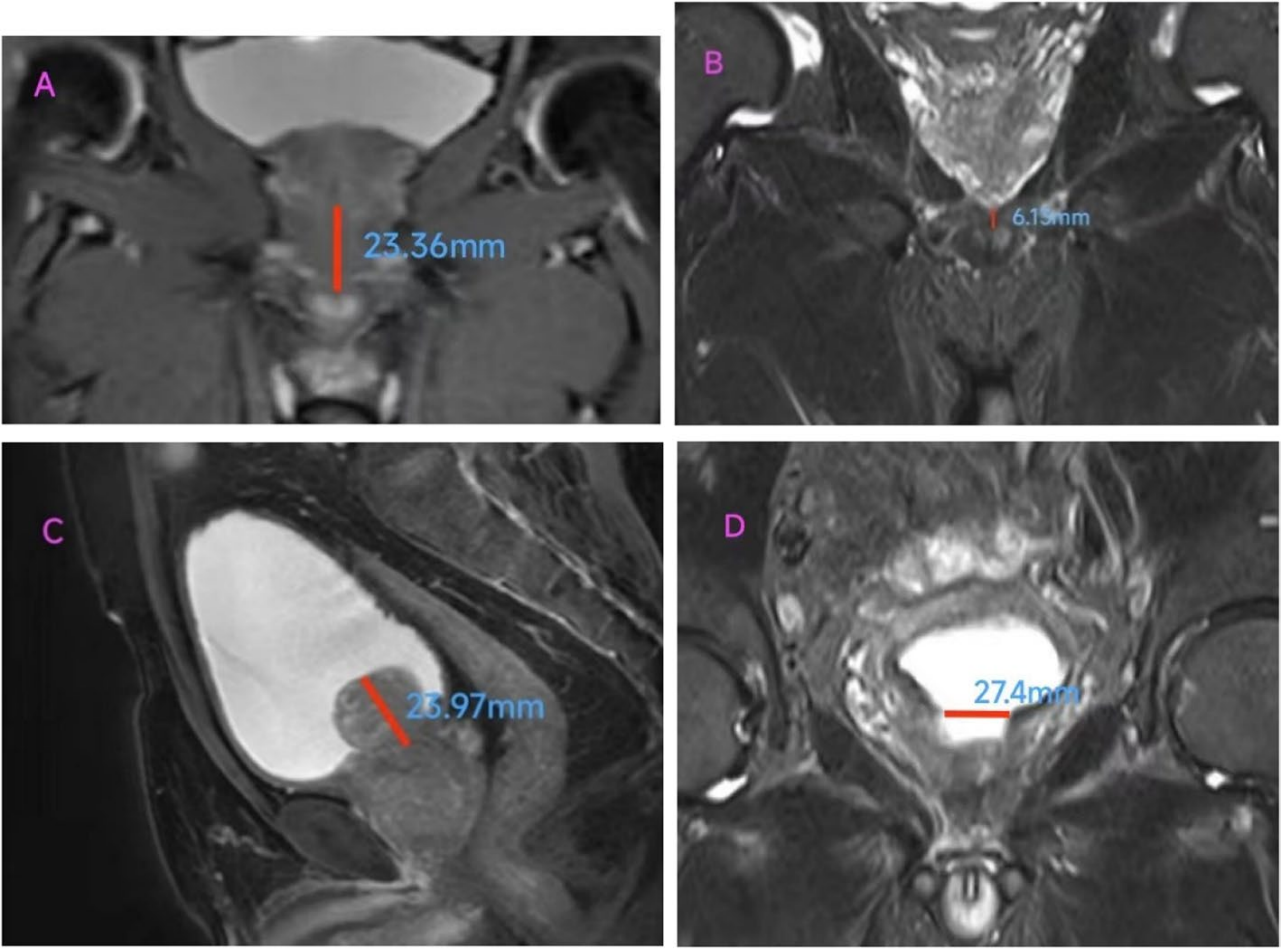
Magnetic resonance measurement parameters of prostate anatomy. **A** the longest MUL **B** the shortest MUL **C** the  the III level IPP **D** the bladder neck diameter

1.5T high field strength superconducting mpMRI instrument (Siemens, Germany) was used to examine the patients. Images were obtained in 2-mm slices with T2-weighted sequences of the entire pelvis in the axial, sagittal and coronal views. MUL was estimated in the midline coronal plane. MUL was defined as the distance from the prostate apex to the urethral entry into the penile bulb was measured on T2-weighted sequences [11, 12] (Fig. 1: A and B). Prostate volume was calculated using the formula for an ellipse (length * width * height * 0.52), the length and width were measured on axial view, while the height was measured on sagittal view. IPP was measured by the vertical distance from the tip of the protruding prostate to the base of the urinary bladder in the sagittal plane of mpMRI (Fig. 1C), which reflected the maximum longitudinal length of the prostate as suggested by Nose et al. [13]. We divided the patients into three groups based on their degree of IPP (I, < 5 mm; II, 5–10 mm; III, > 10 mm). Bladder neck diameter was measured from the bladder opening to the maximum diameter of the prostate in the coronal plane (Fig. 1D).

### LRP

After successful anesthesia, the patient’s head was placed in a low supine position and routinely disinfected and covered. Hip pillow, F16 foley catheter indentured. The anterior sheath of rectus abdominis was separated and exposed in the retropubic space along the bilateral rectus abdominis muscle. A 5-mm and 10-mm Trocar were placed about 2 cm below the incision and at the outer edge of rectus abdominis, respectively, guided by fingers. Satisfaction with pneumoperitoneum was then established with instrumental sutures to reduce the incision and 10 mm Trocar was placed. After exposing the anterior bladder space, a sharp ultrasound knife separation was used to remove the covering adipose tissue, exposing the pelvic fascial space. Carefully cut the dorsal side of the bladder neck, and carefully separate the base of the prostate, so that the bladder neck and the prostatic urethral junction is fully exposed after incision, continue to extend the base of the prostate to separate the seminal vesicle gland. Bilateral seminal vesicles and ampulla of vas deferens were separated from the posterior part of prostate and the surface of Dieldahl fascia. The shallow surface of the Dieldahl fascia is free of space between the rectum and the prostate. The left side of the prostate is free within the fascia, and the right side of the prostate is free within the fascia. The deep dorsal venous plexus of the penis was severed by ultrasound knife. The posterior urethra was severed at the distal end of the prostatic tip. Remove the prostate gland with the seminal vesicle specimen. The bladder neck was shaped, urethra and bladder neck were anastomosed with 3 − 0 barb line of five-eighth arc, F20 foley three-cavity catheter was inserted, and 20 ml of water was injected into the balloon. The neck of the bladder is suspended from behind the pubis. After surgical wound hemostasis, the deep dorsal vein of the penis was still bleeding, and the bleeding stopped after filling with hemostatic gauze. The median incision of the lower abdomen was opened to remove the specimen. A plasma drainage tube was retained in the right side of the pelvic cavity through the puncture hole of the right rectus abdominis muscle, and the incision was closed after fixation.

### Statistical analysis

Quantitative data were expressed as means. Results are reported as mean ± SD. Univariate analysis was performed on all variables, then multivariate logistic regression analyses were performed to determine predictive factors associated with different times of recovery of UC after LRP. The proportion of UC recovery was compared at 3 and 12 mo after LRP. Odds ratio (OR) and 95% confidence interval (CI) were determined. All statistical analyses were performed using SPSS Statistics version 22 (IBM, Armonk, NY, USA). Two-sided *P* < 0.05 was considered statistically significant.

## Results

We enrolled 129 PCa patients. Median follow-up time was 36 (20–58) mo. The mean patient age was 68 ± 6.3 years. There were 36 (27.9%) patients with a history of transurethral resection of the prostate. Mean base PSA was 34.1 ± 34.3 ng/mL, < 10 ng/mL in 26 cases (20.2%), 10–20 ng/mL in 31 cases (24.0%) and > 20 ng/mL in 72 cases (55.8%), respectively. For clinical T stage, ≤ T2a in 20 cases (15.5%), T2b in 29 cases (22.5%) and ≥ T2c in 80 cases (62.0%), respectively. For ISUP grade groups, grade I in 21 cases (16.3%), grade II in 28 cases (21.7%), grade III in 29 cases (22.5%), grade IV in 20 cases (15.5%)and grade V in 31 cases (24%), respectively. For IPP classification, grade I in 121 cases (93.8%), grade II in 6 cases (4.7%) and grade III in 2 cases (1.6%), respectively. Mean MUL was 14.51 ± 3.19 mm. All tumor characteristics, mpMRI parameters and clinical data are shown in Table 1. The immediate UC rates was 27.9% (*n* = 36). UC improved over time, with 54.3% (*n* = 70), 75.2% (*n* = 97) and 88.4% (*n* = 114) achieving UC at 3 mo (short term), 6 and 12 mo (long term), respectively.

The univariate and multivariate associations between UC recovery and predictors at 3 and 12 mo after LRP are shown in Tables 2 and 3 (An additional movie file shows this in more detail [see Additional file 1]). Univariate analysis suggested that MUL was the only variable associated with UC recovery at immediate (OR 1.51; 95% CI 1.26–1.82, *P* < 0.001) ,3 mo after LRP (OR 1.68; 95% CI 1.39–2.02, *P* < 0.001), 6 mo after LRP (OR 1.43; 95% CI 1.22–1.68, *P* < 0.001) and 12 mo after LRP (OR 1.34; 95% CI 1.11–1.62, *P* = 0.002). Meanwhile, multivariate analysis suggested that MUL was a significant independent predictor of UC recovery at immediate [OR 1.77; 95% CI 1.37–2.28; *P* < 0.001], 3 mo (OR 2.25; 95% CI 1.63–3.10; *P* < 0.001), 6 mo (OR 1.47; 95% CI 1.20–1.80; *P* < 0.001) and 12 mo (OR 1.50; 95% CI 1.11–2.04; *P* = 0.009) after surgery.

**Table 1:** Patient Demographic and Clinical Characteristics

Characteristic (*n* = 129)	Value
Age (years, mean ± SD )	68 ± 6.3
BMI^1^ (kg/m^2^, mean ± SD)	24.1 ± 2.8
Smoking history (n, %)	
No	42 (32.6)
Yes	87 (67.4)
Complicated with chronic diseases^2^ (n, %)	
No	59 (45.7)
Yes	70 (54.3)
Transurethral surgery (n, %)	
No	93 (72.1)
Yes	36 (27.9)
Neoadjuvant therapy^3^ (n, %)	
No	87 (67.4)
Yes	42 (32.6)
PSA group^4^ (ng/ml, n, %)	
<10	26 (20.2)
10–20	31 (24.0)
>20	72 (55.8)
Clinical T stage (n, %)	
< T2b	20 (15.5)
T2b	29 (22.5)
> T2b	80 (62.0)
D’Amico risk group (n, %)	
Low	5 (3.9)
Intermediate	13 (10.1)
High	111 (86.0)
ISUP group^5^ (n, %)	
1	21 (16.3)
2	28 (21.7)
3	29 (22.5)
4	20 (15.5)
5	31 (24.0)
Time of operation (minutes, mean ± SD)	248.4 ± 80.7
Pelvic lymph node dissection (n, %)	
No	25 (19.4)
Yes	104 (80.6)
Preserve NVB^6^ (n, %)	
No	120 (93.0)
Yes	9 (7.0)
IPP^7^ (n, %)	
I	121 (93.8)
II	6 (4.7)
III	2 (1.6)
Prostate volume (ml, mean ± SD)	38.86 ± 14.02 (29.26, 48.20)
MUL^8^ (mm, mean ± SD)	14.51 ± 3.19
Bladder neck diameter^9^(mm, mean ± SD)	15.1 ± 4.9

^1^: Body Mass Index; ^2^: including hypertension, diabetes, hyperlipidaemia, cardiopathy, chronic nephrosis, chronic liver disease and so on; ^3^: Neoadjuvant therapy means preoperative endocrinotherapy; ^4^: Prostate specific antigen; ^5^: International Society of Urological Pathology; ^6^: Neurovascular Bundles; ^7^: Intravesical prostatic protrusion, I II III mean protrusion depth are <5 mm, 5–10 mm,>10 mm, respectively; ^8^: Membranous urethral length; ^9^ Bladder neck diameter means the maximum length of the bladder neck opening

**Table 2:** Univariable and Multivariable Analyses of short-term (at 3months) Urinary Continence after RP.

Analysis and Variable		Univariable Analysis Odds Ratio* *P* Value		Multivariable Analysis Odds Ratio* *P* Value				
Age		0.94(0.88, 0.99)	0.03*			0.91(0.81, 1.01)		0.08
BMI		0.95(0.83, 1.07)	0.38			1.04(0.85, 1.27)		0.73
Smoking history								
No		Ref	Ref			Ref		Ref
Yes		1.12(0.54, 2.34)	0.77			0.62(0.17, 2.19)		0.45
Complicated with chronic diseases								
No		Ref	Ref			Ref		Ref
Yes		0.94(0.47, 1.88)	0.85			0.75(0.23, 2.44)		0.63
TURP history								
No		Ref	Ref			Ref		Ref
Yes		1.26(0.58, 2.74)	0.56			4.00(0.69, 23.30)		0.12
Neoadjuvant therapy								
No		Ref	Ref			Ref		Ref
Yes		0.89(0.42, 1.87)	0.77			0.33(0.08, 1.39)		0.13
PSA group								
<10		Ref	Ref			Ref		Ref
10–20		0.59(0.20, 1.69)	0.32			1.31(0.19, 8.78)		0.78
>20		0.74(0.30, 1.85)	0.52			4.61(0.70, 30.19)		0.11
Clinical T stage								
≤T2a		Ref	Ref			Ref		Ref
T2b		1.56(0.48, 5.00)	0.46		10.15(1.22, 84.34)			0.03*
≥T2c		0.82(0.31, 2.19)	0.69			1.76(0.29, 10.82)		0.54
D’Amico risk group								
Low		Ref	Ref			Ref		Ref
Intermediate	0.83(0.07, 10.60)		0.89			0.46(0.01, 17.79)		0.67
High		0.26(0.03, 2.35)	0.23			0.12(0.00, 4.87)		0.26
ISUP group								
1		Ref	Ref			Ref		Ref
2		0.62(0.18, 2.08)	0.44			0.36(0.06, 2.33)		0.28
3		0.43(0.13, 1.42)	0.16			0.07(0.01, 0.62)		0.02*
4		0.40(0.11, 1.45)	0.16			0.09(0.01, 1.01)		0.05
5		0.29(0.09, 0.95)	0.04*			0.17(0.02, 1.18)		0.07
Time of operation		1.00(1.00, 1.00)	0.69			1.00(0.99, 1.00)		0.16
Pelvic lymph node dissection								
No		Ref	Ref			Ref		Ref
Yes		0.75(0.31, 1.82)	0.52			1.02(0.23, 4.46)		0.98
Preserve NVB								
No		Ref	Ref			Ref		Ref
Yes		0.66(0.17, 2.59)	0.54			0.57(0.07, 4.54)		0.59
PP		0.88(0.30, 2.58)	0.81			0.40(0.05, 3.12)		0.38
Prostate volume		0.99(0.97, 1.02)	0.59			0.99(0.94, 1.04)		0.63
MUL		1.68(1.39, 2.02)	<0.001*			2.25(1.63, 3.10)	<0.001*	
Bladder neck diameter		0.96(0.89, 1.03)	0.26			1.03(0.89, 1.20)		0.69

**Table 3:** Univariable and Multivariable Analyses of long-term Urinary Continence (at 12months) after RP.

Analysis and Variable		Univariable Analysis Odds Ratio* *P* Value		Multivariable Analysis Odds Ratio* *P* Value		
Age		0.97(0.88, 1.06)	0.49		1.00(0.87, 1.14)	0.98
BMI		0.84(0.69, 1.02)	0.08		0.79(0.60, 1.04)	0.10
Smoking history						
No		Ref	Ref		Ref	Ref
Yes		1.44(0.48, 4.37)	0.52		1.37(0.27, 6.87)	0.70
Complicated with chronic diseases						
No		Ref	Ref		Ref	Ref
Yes		1.08(0.37, 3.18)	0.89		1.44(0.33, 2.18)	0.63
TURP history						
No		Ref	Ref		Ref	Ref
Yes		1.07(0.32, 3.62)	0.91		2.24(0.28, 17.67)	0.45
Neoadjuvant therapy						
No		Ref	Ref		Ref	Ref
Yes		2.08(0.55, 7.81)	0.28		3.04(0.36, 25.96)	0.31
PSA group						
<10		Ref	Ref		Ref	Ref
10–20		0.78(0.12, 5.05)	0.79		2.93(0.17, 50.65)	0.46
>20		0.52(0.11, 2.53)	0.42		1.32(0.11, 15.19)	0.83
Clinical T stage						
≤T2a		Ref	Ref		Ref	Ref
T2b		-	-		-	-
≥T2c		-	-		-	-
D’Amico risk group						
Low		Ref	Ref		Ref	Ref
Intermediate		-	-		-	-
High		-	-		-	-
ISUP group						
1		Ref	Ref		Ref	Ref
2	1.37(0.18, 10.60)		0.76		3.00(0.25, 36.33)	0.39
3		0.66(0.11, 3.98)	0.65		0.93(0.08, 10.68)	0.95
4		0.95(0.12, 7.46)	0.96		2.25(0.16, 31.02)	0.54
5		0.55(0.10, 3.13)	0.50		1.11(0.12, 10.48)	0.93
Time of operation		1.00(0.99, 1.01)	0.57		1.00(0.99, 1.01)	0.39
Pelvic lymph node dissection						
No		Ref	Ref		Ref	Ref
Yes		0.61(0.13, 2.89)	0.53		1.56(0.19, 12.62)	0.68
Preserve NVB						
No		Ref	Ref		Ref	Ref
Yes		-	-		-	-
IPP		-	-		-	-
Prostate volume		1.01(0.97, 1.05)	0.72		1.02(0.95, 1.09)	0.66
MUL		1.34(1.11, 1.62)	0.002*		1.50(1.11, 2.04)	0.009*
Bladder neck diameter		1.02(0.91, 1.34)	0.76		1.09(0.88, 1.36)	0.43

## Discussion

Cancer control, preservation of erectile function and UC recovery are the optimal trifecta outcomes after RP [4]. The preoperative or intraoperative factors affecting UC recovery after RP are unclear in the literature, and controversy exits surrounding them [14]. In our current study, marked by 3 and 12 mo postoperatively, we incorporated 17 parameters to analyze their relationship with short- and long-term UC post-LRP. We found that MUL was the only significant predictor for short and long term UC recovery.

First, compared to open RP (ORP) or RARP, the functional results of short and long term UC recovery in our single center were similar to those of other studies [10, 13, 15–17]. Anastasios D. et al. suggested that the difference in the rate of UI after LRP (17%) and RARP (6%) as well as time to UC recovery did not reach the significance at 3 or 12 mo [18]. However, a multi-institutional randomized controlled trial (RCT) [17] showed better UC recovery at 3 mo after RARP, including no pads in 30% of patients compared to 17% in the LRP group. The authors suggested that early UC recovery was associated with better three-dimensional vision and greater dexterity, and that LRP was performed by more-experienced surgeons, which strengthens the validity of the UC recovery findings of this RCT. In contrast, ORP and RARP did not achieve similar results for UC recovery. A large, prospective, controlled, nonrandomized trial showed that 366 men (21.3%) were incontinent after RARP, as were 144 (20.2%) after ORP at 12 mo, and there was no significant difference [16].

Second, some studies [19, 20] that have been published suggest a longer mean time to UC recovery for patients with previous TURP. These patients who have received TURP before LRP usually have lower urinary tract symptoms (LUTS), especially difficulty urinating. Furthermore, the proposed hypothesis that previous TURP leads to worse outcomes in patients undergoing RP for periprostatic inflammation and fibrosis [19]. However, in the present study, we did not find the prior TURP was associated with short and long term UC recovery, Teber D’s study [21] also showed no impact on postoperative UC recovery. These studies believed that tissue separation and adhesion and bladder neck reconstruction are more difficult due to TURP, leading RP might be technically more difficult to perform. If the LRP is performed 3 months after TURP, or if the surgical method is improved, the results of LRP following TURP are indifferent from non-TURP patients [22].

Third, the membranous urethra (MU) is located between the apex of the prostate and the bulbar urethra, which is surrounded by the external urethral sphincter, and constitutes one of the three parts of the anatomical upper urethral stricture. Studies have proven that longer MUL sparing has been recommended to achieve better functional urethral length and shown to improve UC recovery [23, 24]. MUL was preoperatively measured by mpMRI. Longer MUL may lead to more functional urethral retention during surgery, which helps to control urine flow [25]. In addition, urethral sphincter protection is the key factor in UC recovery. The longer MUL increased the safe distance between the prostatic apex and urethral sphincter, and avoided damage of the urethral sphincter [26]. In the present study, MUL was significantly correlated with UC recovery after LRP at the four time points, which means that MUL is an independent predictor for UC recovery post-LRP, and patients with longer MUL will have earlier recovery of short and long term UC recovery. Similarly, Lamberg et al. [27] included 586 PCa patients and demonstrated that longer coronal MUL improved the odds of post-RP UC recovery at 3, 6 and 12 mo. We also measured MUL by coronal mpMRI, because most studies are measured at this level. Furthermore, a recent meta-analysis [28] suggested that the measurement method (sagittal, coronal or both/averaged) did not influence the results, and pooled analysis showed that greater MUL was prognostic for regaining UC recovery at 3 mo. Consequently, there is no doubt that MUL will improve the UC recovery post-LRP.

In the present study, age and IPP were not significant predictors for UC recovery, which differed from prior studies [29, 30]. Interestingly, because of the lack of early PCa screening, PCa patients in China are seem to at an older age for surgery than in western countries. The mean age was 68 ± 6.3 years in our study, and 72.9% of patients were > 65 years, which is older than previous study [31]. In our study, the sample size of IPP grade II and III was small and IPP is generally related to benign prostatic hyperplasia, and these patients will undergo TURP before RP, which further reduces the number of patients with high-graded IPP and statistical errors may occur. In contrast, Lee et al. [13] observed that nonsignificant IPP (< 5 mm) markedly improved UC recovery compared with significant IPP (> 5 mm) at 1, 3, 6 and 12 mo postoperatively. In this paper, we included a total of 17 parameters. considering the importance of the included variable factors in the prediction of urinary incontinence after LRP in practice, all of them were included in the multivariate analysis in the subsequent multi-factor logistics regression analysis.

Finally, our study had some limitations. First, the sample size was small and may not reflect the real-world situation, and this may explain why we did not find more significant variables. Thus, a larger sample and multi-center clinical data are still needed in a follow-up study. Second, the data were collected retrospectively, which can lead to recall bias, and there was no comparison made among these patients to analyze the factors predicting continence rates in this study. Thirdly, although these operations in our study were performed by the same surgeon, the heterogeneity of different surgeons’ experiences and skills need not be considered, and patients’ postoperative recovery is also related to the experiences and skills of the surgeon.

## Conclusions

Our study found that MUL is an independent risk factor for UC recovery after LRP. This means that before surgery, by reading the radiograph and measuring the length of the patient’s MUL, we can roughly judge the recovery time of postoperative UC of the patient, communicate with the patient in advance, so as to alleviate the patient’s postoperative anxiety and establish a good doctor-patient relationship. This is a single-center data from the Chinese Cancer Hospital, which is instructive for the analysis of urinary incontinence after RP in the Chinese population.

## Data Availability

No datasets were generated or analysed during the current study.
